# A High Throughput HPLC-MS/MS Method for Antihypertensive Drugs Determination in Plasma and Its Application on Pharmacokinetic Interaction Study with Shuxuetong Injection in Rats

**DOI:** 10.1155/2019/7537618

**Published:** 2019-08-06

**Authors:** Juan Wei, Wenjuan Ma, Guangzhe Yao, Qi Jia, Xuejing Cheng, Huizi Ouyang, Yanxu Chang, Xiaopeng Chen, Jun He

**Affiliations:** Tianjin State Key Laboratory of Modern Chinese Medicine, Tianjin University of Traditional Chinese Medicine, Tianjin, 301617, China

## Abstract

A high-throughput HPLC-MS/MS method was developed and validated for the determination of four antihypertensive drugs including metoprolol tartrate, hydrochlorothiazide, nifedipine, and valsartan in rat plasma. The Sprague-Dawley rats were randomly divided into three groups: A Group: gastric-administration of metoprolol tartrate, hydrochlorothiazide, nifedipine, or valsartan; B Group: a single intravenous injection of SXT, then dosing as the A group; C Group: daily injection of SXT through the tail vein for 8 consecutive days and dosing as the A group on the eighth day. For metoprolol tartrate and valsartan, blood samples were collected before administration and at time points 0.03, 0.08, 0.17, 0.25, 0.5, 1, 2, 4, 6, 8, 10, 12, and 24 h from the fossa orbitalis vein. For hydrochlorothiazide and nifedipine, the time points were 0, 0.08, 0.17, 0.25, 0.5, 0.75, 1, 2, 4, 6, 8, 10, 12, and 24 h. The plasma samples containing different individual antihypertensive drug were mixed and prepared by protein precipitation with methanol. The chromatographic separation was performed on an Agilent Eclipse Plus C18 column (2.1 mm×100 mm, 3.5 *μ*m) using gradient elution with mobile phase consisting of acetonitrile and water (containing 0.1% formic acid). The flow rate was 0.3 mL/min. The detection was accomplished on a tandem mass spectrometer with an electrospray ionization (ESI) source by multiple reaction monitoring (MRM) in both positive and negative modes. The method was successfully applied to a pharmacokinetic interaction study of Shuxuetong injection on the antihypertensive drugs. The results suggested that SXT could increase the total amount of metoprolol tartrate and nifedipine in plasma and showed little influence on the pharmacokinetic behaviors of hydrochlorothiazide and valsartan.

## 1. Introduction

Traditional medicine has become widely used in the last decade. According to a recent survey, about 70% of the world's population (including 40% of Americans) use traditional medicine as supplements or alternative medicine [1]. Since the concomitant application of traditional and western medicines has become a trend, herb-drug interactions have been extensively investigated worldwide, and an increasing interest in clinical herb-drug interactions blooms [2, 3]. The interaction between traditional and western medicine refers to the changes in pharmacodynamic and pharmacological effects caused by the combination or sequential usage, including pharmacokinetic interactions and pharmacodynamic interactions. Understanding of the pharmacokinetic basis of the interaction is significant for guiding clinical rational drug use, improving clinical treatment effects, and reducing toxic and side effects [4].

Shuxuetong injection (SXT) is traditionally used in Chinese medicine to treat “blood stasis and stagnation” (yuxue yuzhi), particularly in patients with cerebral and myocardial infarction [5]. SXT is extracted from leech (*Whitmania pigra*) and earthworm (*Pheretima aspergillum*). The main components of SXT are carbohydrates, amino acids, peptides, and terpenoids [6, 7]. It is widely used in the treatment of ischemic stroke, coronary heart disease, cerebral ischemia, diabetic peripheral vascular disease and chronic renal failure [8]. According to publications, SXT and antihypertensive western drugs rank top 20 in combination therapy of traditional and western medication in ischemic stroke treatment [9]. Because of combination therapy, herb-drug interaction risk may emerge clinically.

Based on clinical conditions [9], an HPLC-MS/MS method was developed to four kinds of antihypertensive drugs in rat plasma in the present study. The four types of drugs are metoprolol tartrate (*β*1-selective adrenergic blocker), hydrochlorothiazide (thiazide diuretics), nifedipine (calcium antagonist), and valsartan (angiotensin II receptor antagonist), which stand for the four main treatment mechanism of antihypertensive drugs. This method was applied to the pharmacokinetic interaction study of SXT on antihypertensive drugs. Dosing four kinds of antihypertensive drugs separately, individual rat plasma was mixed together and an analytical method for the four drugs in one injection was developed. This method allows for the analysis of the above four different kinds of antihypertensive drugs in one injection which provides high efficiency and high throughput. It is the first investigation on the pharmacokinetic interactions of SXT on the antihypertensive drugs. The results would provide a theoretical basis for combined medication of SXT and antihypertensive drugs clinically.

## 2. Materials and Methods

### 2.1. Chemicals and Reagents

**Figure 1:**
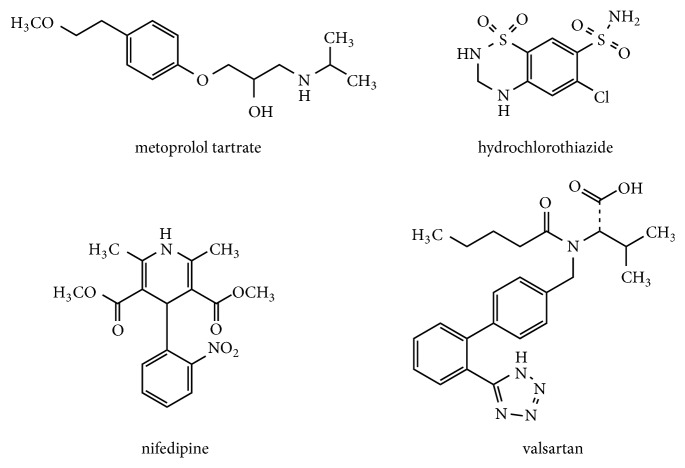
Chemical structures of four components.

Acetonitrile and methanol of HPLC grade were purchased from Merck (Darmstadt, Germany). Formic acid of HPLC grade was obtained from ROE (Newark, USA). Ultrapure water was obtained through a Milli-Q system. Metoprolol tartrate, hydrochlorothiazide, nifedipine, valsartan and methyclothiazide were purchased from the China National Institutes for Food and Drug Control. Shuxuetong injection was provided by Mudanjiang Youbo Pharmaceutical Co. Ltd. (Heilongjiang, China). The structures of the analytes were shown in Figure 1.

### 2.2. LC-MS/MS Conditions

Chromatographic separation was achieved on an Agilent Eclipse Plus C18 column (2.1mm×100 mm, 3.5*μ*m) using an Agilent 1200 HPLC system equipped with the quaternary pump, auto-sampler, column oven and degasser. The mobile phase containing 0.1% (v/v) formic acid (A) and acetonitrile (B) was at a flow rate of 0.3 mL/min. The gradient program was as follows: 0-4.0 min, 6-50% B, 4.0-5.0 min, 50%-80% B, 5.0-7.0 min, 80%-95% B, 7.0-10.0 min, and 95%-95% B. The separation temperature was set at 30°C and the sample injection volume was 5 *μ*L.

MS detection was operated on an Agilent 6430 triple quadrupole mass spectrometer coupled with electrospray ionization (ESI) source. The mass spectrometer was set in both positive and negative ion mode. The optimum MS parameters were maintained as follows: nebulizer, 30 psi; drying gas (N2) flow rate, 10 L/min; capillary temperature, 320°C. The MRM parameters of four drugs and IS were shown in Table 1.

### 2.3. Preparation of Calibration Standards and Quality Control Samples

The four antihypertensive drugs were weighed separately. Metoprolol tartrate, hydrochlorothiazide and valsartan were dissolved in 50% methanol while nifedipine in methanol to make the stock solutions (1 mg/mL). The working solutions of the four drugs were obtained by mixing and diluting the stock solutions with 50% methanol to obtain concentrations of 20-10000 ng/mL for metoprolol tartrate, 25-12500 ng/mL for hydrochlorothiazide, 5-2500 ng/mL for nifedipine, and 50-25000 ng/mL for valsartan. Methyclothiazide was dissolved in methanol to produce the IS solution with a concentration of 1 *μ*g/mL.

The calibration standard solutions were prepared by adding appropriate amounts of working solutions into 200 *μ*L blank rat plasma to give nominal concentration range of 8-4000 ng/mL for metoprolol tartrate, 10-5000 ng/mL for hydrochlorothiazide, 2-1000 ng/mL for nifedipine, and 20-10000 ng/mL for valsartan.

Quality control (QC) samples at low, medium and high concentration were prepared in the same manner at 20, 400 and 4000 ng/mL for metoprolol tartrate, 25, 500 and 5000 ng/mL for hydrochlorothiazide, 5, 100 and 1000 ng/mL for nifedipine, and 50, 1000 and 10000 ng/mL for valsartan. All the solutions were stored at 4°C until analysis.

### 2.4. Sample Preparation

Rat plasma samples were thawed to room temperature before use. 200 *μ*L plasma sample (50 *μ*L for each of the four antihypertensive drugs after oral administration in rats, respectively), 20 *μ*L methyclothiazide (1 *μ*g/mL) and 20 *μ*L 50% methanol were added into a 1.5 mL centrifuge tube and mixed by vortexing. The mixed sample was precipitated with 800 *μ*L of methanol and vortexed for 3 min. The tubes were then centrifuged at 14,000 rpm for 10 min to obtain clean supernatants which were evaporated to dryness under a gentle stream of nitrogen at room temperature. The obtained residue was dissolved in 100 *μ*L of 50% methanol and centrifuged at 14,000 rpm for another 10 min. Finally, 5 *μ*L supernatant was injected into the LC-MS/MS system for analysis.

### 2.5. Method Validation

The method was validated in specificity, linearity, accuracy, precision, extraction recovery, matrix effect, and stability according to the USFDA guidelines.

#### 2.5.1. Specificity

The specificity of the method was assessed by comparing chromatograms of blank plasma samples from six different rats, blank plasma spiked with the four drugs and IS, and rat plasma samples obtained at 1 h after administration. The interference from plasma matrix was estimated by the proposed LC-MS/MS condition.

#### 2.5.2. Linearity

Calibration curves were obtained using a series of calibration samples and constructed by plotting the peak area ratio of analyte to IS (y) against the concentration of the analyte in spiked plasma samples (x). 1/x^2^ was the weighting factor. The lower limit of quantification (LLOQ) was determined at a signal-to-noise ratio of about 10 by analyzing the standard spiked plasma samples.

#### 2.5.3. Precision and Accuracy

The accuracy and precision (both intra- and inter-day) were estimated by analyzing QC samples. Six replicates of QC samples at low, medium and high concentration levels (20, 400 and 4000 ng/mL for metoprolol tartrate; 25, 500 and 5000 ng/mL for hydrochlorothiazide; 5, 100 and 1000 ng/mL for nifedipine; 50, 1000 and 10000 ng/ml for valsartan) were analyzed on three consecutive runs. The accuracy was expressed as a relative error (RE) and the data was required to be within ± 15%. The precision was assessed as relative standard deviation (RSD) and should be less than 15%.

#### 2.5.4. Extraction Recovery and Matrix Effect

The extraction recovery and matrix effect of four drugs were calculated at three QC concentration levels in six replicates. The extraction recovery was calculated as the ratio of the peak area of the extracted samples compared with the postextraction spiked samples. The matrix effect was obtained by comparing the peak areas of the post-extraction spiked samples versus standard solutions at the same concentration.

#### 2.5.5. Stability

The stability of the four drugs in rat plasma was measured by analyzing QC samples at a variety of conditions: room temperature for 4 h, in autosampler after post-treatment for 12 h, −70°C for two weeks, and three freeze-thaw cycles. Three replicates were investigated at low, medium and high concentration at each storage conditions.

#### 2.5.6. Animals and Pharmacokinetic Study

Seventy-two male Sprague-Dawley rats (230 ± 20 g) were used for pharmacokinetic study. The rats were raised with standard diet and water in a stable environment. The temperature was 23-26°C and relative humidity was 40-60%. After a week of acclimation, the animals were fasted 12 h before the experiment and allowed free access to water during the experiment. In order to explore the effects of single and multiple dosing of SXT on the pharmacokinetics of four antihypertensive drugs, the rats were randomly divided into three groups. A Group: gastric-administration of metoprolol tartrate, hydrochlorothiazide, nifedipine or valsartan at a dose of 41.67 mg/kg, 10.42 mg/kg, 4.17mg/kg or 4.17 mg/kg, respectively. The dosage was converted based on clinical usage. B Group: a single intravenous injection of SXT (0.625 mL/kg) and then dosing as the A group. C Group: injection of SXT (0.625 mL/kg) through the tail vein daily for 8 consecutive days and dosing as the A group on the eighth day. At the time of administration, metoprolol tartrate was dissolved in water and others were dissolved in 0.5% CMC-Na. For metoprolol tartrate and valsartan, blood samples (0.2 mL) of each rat were collected before administration and at time points 0.03, 0.08, 0.17, 0.25, 0.5, 1, 2, 4, 6, 8, 10, 12, and 24 h from the fossa orbitalis vein into heparinized tubes after administration. For hydrochlorothiazide and nifedipine, the time points were 0, 0.08, 0.17, 0.25, 0.5, 0.75, 1, 2, 4, 6, 8, 10, 12, and 24 h. Blood samples were transferred into a heparinized microcentrifuge tube and centrifuged at 7000 rpm for 10 min to get the plasma samples. All the plasma samples were stored at -70°C until analysis. The animal studies described in this paper were approved and conducted in accordance with the guidelines of the Laboratory Animal Ethics Committee of Tianjin University of Traditional Chinese Medicine. The rat plasma concentration-time data were computed by the software “Drug and Statistics 2.0” (DAS 2.0).

## 3. Results and Discussion

### 3.1. Optimization of LC-MS/MS Method

The mobile phase is a critical factor in achieving satisfactory chromatographic retention time, symmetric peak shape and high response for all the drugs and IS. Acetonitrile was selected as the mobile phase because of its powerful elution ability. The response of four drugs and IS was improved with the addition of 0.1% formic acid into water. The analytes had good retention time, peak symmetry and appropriate ionization with gradient elution.

In the precursor ion full-scan spectra, the most abundant ions of hydrochlorothiazide, valsartan and methyclothiazide (IS) were deprotonated molecules as [M–H]^−^. Metoprolol tartrate and nifedipine had good response in positive mode and gave protonated ions [M+H]^+^ as major peaks. The transitions m/z 295.8/205.2 for hydrochlorothiazide, 434.2/179.0 for valsartan, 357.9/321.8 for methyclothiazide (IS), 268.1/121.0 for metoprolol tartrate, and 347.1/254.1 for nifedipine were chosen for the quantification studies.

### 3.2. Method Validation

#### 3.2.1. Specificity

**Figure 2:**
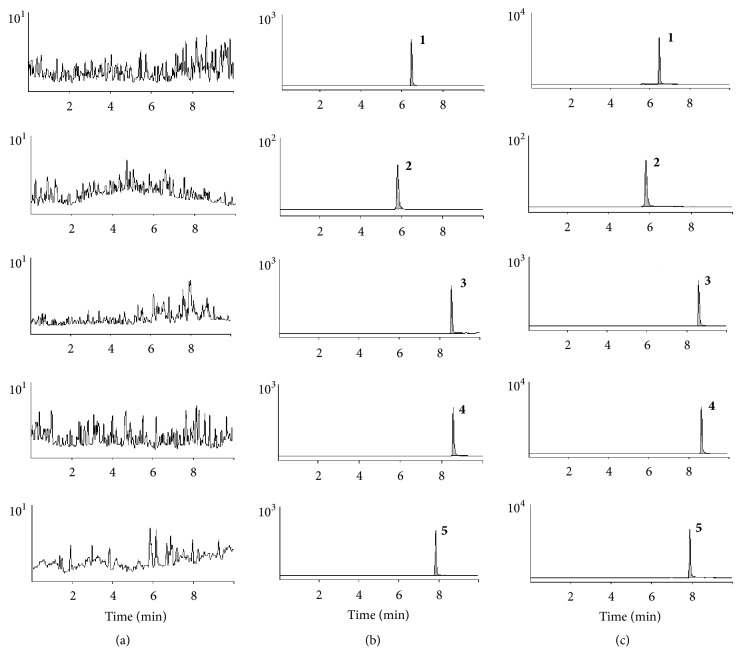
MRM chromatograms of metoprolol tartrate** (1)**, hydrochlorothiazide** (2)**, nifedipine** (3)**, valsartan** (4)**, and IS** (5)**, (a) blank plasma; (b) blank plasma spiked with the analytes and IS; (c) 1 h plasma sample after oral administration of four antihypertensive drugs, respectively.

Specificity was evaluated by analyzing six different blank plasma samples. The representative chromatograms of blank plasma (A), blank plasma spiked with four drugs and IS (B), and rat plasma sample collected at 1 h after oral administration of four drugs (C) were shown in Figure 2. The retention time of metoprolol tartrate, hydrochlorothiazide, nifedipine, valsartan, and methyclothiazide were 6.47 min, 5.81 min, 8.59 min, 8.60 min, and 7.86 min, respectively. As shown in Figure 2, there was no significant interference at the corresponding analytes retention time.

#### 3.2.2. Linearity

The correlation coefficients (r) of the generated calibration curves for each drug were ≥ 0.9906 in the plasma samples. Thus, it shows a good linear response over the range 8-4000 ng/mL for metoprolol tartrate, 10-5000 ng/mL for hydrochlorothiazide, 2-1000 ng/mL for nifedipine and 20-10000 ng/mL for valsartan. The regression equations, linear ranges, correlation coefficients and LLOQ were presented in Table 2.

#### 3.2.3. Precision and Accuracy

The intra- and inter-day precision at three different concentration levels (low, medium and high) of four drugs were less than 9.1%, while the accuracy ranged from -13.5% to 12.7%. The results of the precision and accuracy meet the criteria of biological sample analysis. The intra- and interday precision and accuracy of four drugs were shown in Table 3.

#### 3.2.4. Extraction Recovery and Matrix Effect

The mean extraction recoveries of four drugs ranged from 86.2% to 111.5% at the three concentration levels. The mean matrix effects of the four drugs were between 67.8% and 108.4%. The results indicated that the efficiency of protein precipitation is acceptable and the endogenous matrix peaks could not affect the quantification of the analytes. The extraction recovery and matrix effect data were summarized in Table 4.

#### 3.2.5. Stability

The results of stability were shown in Table 5. The RSDs of the response were less than 11.7% for all stability tests of metoprolol tartrate, hydrochlorothiazide, nifedipine and valsartan. The data showed that the stability of each drug was acceptable and applicable under the different study conditions.

#### 3.2.6. Application

**Figure 3:**
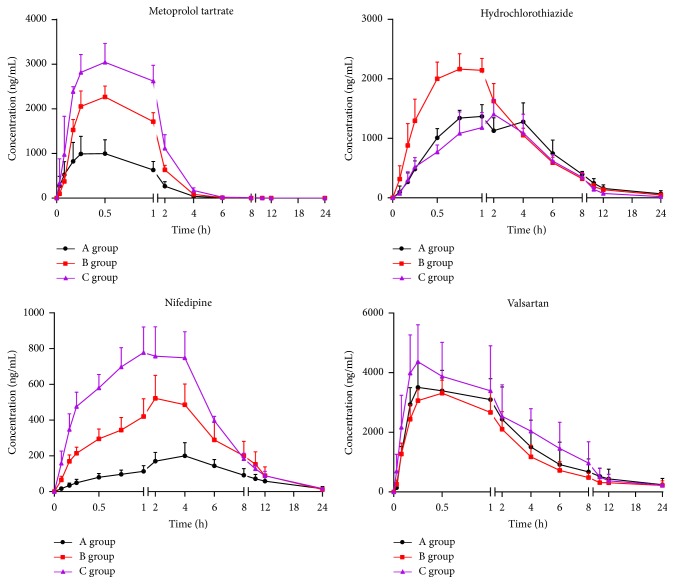
Mean plasma concentration-time curves in different groups following oral administration of metoprolol tartrate, hydrochlorothiazide, nifedipine, and valsartan, respectively (mean ± SD, n=6).

The validated method was applied to the pharmacokinetic study of four antihypertensive drugs (metoprolol tartrate, hydrochlorothiazide, nifedipine and valsartan) in rat plasma. The mean plasma concentration-time curves of four drugs were shown in Figure 3, and the pharmacokinetic parameters were presented in Table 6.

As shown in Figure 3 and Table 6, the C_*max*_ and AUC_(0-24)_ of metoprolol tartrate in group B (2277.29 ± 240.30 ng/mL, 3787.97 ± 283.12 h ng/mL) and C (3045.11 ± 424.22 ng/mL, 5975.49 ± 776.71 h ng/mL) were increased significantly (P≤0.01) compared to group A (1068.55 ± 330.39 ng/mL, 1621.21 ± 464.32 h ng/mL). Nifedipine showed a similar trend. The increase in AUC_(0-24)_ represents the total increase of metoprolol tartrate and nifedipine in plasma, indicating increased bioavailability. The upward trend of half-life (t_*1/2*_) of metoprolol tartrate in group C suggests that the elimination of metoprolol tartrate in rats was postponed after long term intravenous administration of SXT. As reported, metoprolol tartrate is the substrate of CYP2D6 [10] while nifedipine is the substrate of CYP3A4 [11]. Further experiments were carried out on the SXT influence on CYP450 activity (Supplementary materials (available here)). SXT could inhibit the activity of CYP2D6 and CYP3A4. The inhibition on CYP2D6 and CYP3A4 may decrease the metabolism of metoprolol tartrate and nifedipine and therefore increase its total amount in plasma.

The C_*max*_ of hydrochlorothiazide in group A (1515.75 ± 235.11 ng/mL) were decreased (P≤0.01) compared to group B (2292.22 ± 209.37 ng/mL), but had no significant difference (P>0.05) when compared with group C (1406.19 ± 248.74 ng/mL). This shows that single intravenous administration of SXT affects the excretion of hydrochlorothiazide in rats, but this effect disappears with long term SXT administration. Since hydrochlorothiazide is the substrate of P-glycoprotein participating in the excretion process, it is deduced that SXT may affect the activity of P-gp. As for valsartan, no significant difference was observed on pharmacokinetic parameters of the three groups.

#### 3.2.7. Discussion

In the experiment, we evaluated the solubility of the analytes in different solvents. Accroding to their physical and chemical properties, metoprolol tartrate has good solubility in water but others do not have. Hence, hydrochlorothiazide, nifedipine and valsartan were dissolved in 0.5% CMC-Na in the pharmacokinetics study.

## 4. Conclusion

In this study, a high-throughput LC-MS/MS method was established to determine four antihypertensive drugs including metoprolol tartrate, hydrochlorothiazide, nifedipine and valsartan in rat plasma. The method was applied to the pharmacokinetic interaction study of SXT on antihypertensive drugs. SXT could increase the total amount of metoprolol tartrate and nifedipine in plasma, and showed little influence on the pharmacokinetic behaviors of hydrochlorothiazide and valsartan. The mechanisms of pharmacokinetic interaction of SXT on the antihypertensive drugs need to be further investigated. The concomitant application of traditional and western medicines is commonly used clinically. This results provide a theoretical basis for combined medication of SXT and antihypertensive drugs in ischemic stroke treatment.

## Figures and Tables

**Table 1:** Mass spectra properties of the analytes and IS.

Compounds	Precursor Ion (m/z)	Product Ion (m/z)	Frag. (V)	C.E. (V)
metoprolol tartrate	268.1	121.0	95	20
hydrochlorothiazide	295.8	205.2	115	-16
nifedipine	347.1	254.1	91	14
valsartan	434.2	179.0	123	-20
methyclothiazide(IS)	357.9	321.8	135	-3

**Table 2:** Calibration curves, correlation coefficients, linear ranges, and LLOQs of the analytes.

Compounds	Calibration curve	r	Linear range (ng/mL)	LLOQ (ng/mL)
metoprolol tartrate	y = 1.1056 x + 0.0057	0.9962	8.0-4000.0	0.1
hydrochlorothiazide	y = 0.0536 x + 0.0012	0.9906	10.0-5000.0	5.0
nifedipine	y = 0.7370 x + 0.0021	0.9924	2.0-1000.0	1.0
valsartan	y = 0.3144 x + 0.0030	0.9958	20.0-10000.0	0.1

**Table 3:** Precision and accuracy of the analytes in rat plasma (n = 6).

Compounds	Spiked concentration (ng/mL)	Intraday			Interday		
		Measured (ng/mL)	RE (%)	RSD (%)	Measured (ng/mL)	RE (%)	RSD (%)
metoprolol tartrate	20	21.16 ± 0.91	5.8	4.3	21.16 ± 0.64	5.8	3.0
	400	431.98 ± 10.65	8.0	2.5	427.53 ± 12.12	6.9	2.8
	4000	3725.53 ± 113.06	-6.9	3.0	3784.38 ± 153.35	-5.4	4.1
hydrochlorothiazide	25	24.07 ± 1.70	-3.7	7.1	26.07 ± 2.37	4.3	9.1
	500	531.80 ± 28.55	6.4	5.4	524.21 ± 29.44	4.8	5.6
	5000	4551.15 ± 42.31	-9.0	0.9	4510.25 ± 147.90	-9.8	3.3
	5	5.27 ± 0.33	5.5	6.2	5.34 ± 0.30	6.9	5.6
nifedipine	100	100.64 ± 2.47	0.6	2.5	106.67 ± 5.20	6.7	4.9
	1000	909.20 ± 13.18	-9.1	1.5	902.93 ± 43.30	-9.7	4.8
	50	56.37 ± 3.12	12.7	5.5	55.30 ± 1.94	10.6	3.5
valsartan	1000	1074.78 ± 24.16	7.5	2.3	1083.06 ± 23.84	8.3	2.2
	10000	8647.62 ± 294.77	-13.5	3.4	8813.82 ± 376.14	-11.9	4.3

**Table 4:** Extraction recoveries and matrix effects of the analytes (n = 6).

Compounds	Spiked concentration (ng/mL)	Extraction recovery (%)	RSD (%)	Matrix effect (%)	RSD (%)
metoprolol tartrate	20	99.95 ± 2.66	2.7	99.99 ± 1.98	2.0
	400	91.00 ± 1.92	2.1	90.86 ± 1.44	1.6
	4000	95.36 ± 1.08	1.1	91.77 ± 0.63	0.7
hydrochlorothiazide	25	111.50 ± 7.24	6.5	108.41 ± 7.42	6.9
	500	99.77 ± 3.48	3.5	107.96 ± 3.32	3.1
	5000	98.09 ± 2.84	2.9	98.63 ± 2.72	2.8
	5	92.76 ± 5.36	5.8	67.83 ± 5.34	7.9
nifedipine	100	86.20 ± 2.52	2.9	70.52 ± 0.43	0.6
	1000	89.44 ± 2.44	2.7	82.64 ± 0.38	0.5
	50	111.53 ± 7.45	6.7	97.45 ± 6.73	6.9
valsartan	1000	98.55 ± 4.58	4.6	97.73 ± 4.71	4.8
	10000	100.81 ± 2.27	2.3	90.56 ± 1.22	1.3

**Table 5:** Stability of the analytes in rat plasma (n = 3).

Compounds	Spiked Concentration (ng/mL)	room temperature for 4 h		three freeze-thaw cycles		auto-sampler for 12 h		-70°C for 14 days	
		Measured (ng/mL)	RSD (%)	Measured (ng/mL)	RSD (%)	Measured (ng/mL)	RSD (%)	Measured (ng/mL)	RSD (%)
metoprolol tartrate	20	20.95 ± 0.73	3.5	20.05 ± 1.71	8.5	21.42 ± 0.17	0.8	22.12 ± 1.33	6.0
	400	437.09 ± 4.45	1.0	412.18 ± 8.83	2.1	408.30 ± 5.14	1.3	448.14 ± 4.22	0.9
	4000	3939.11 ± 62.55	1.6	3941.70 ± 205.62	5.2	3732.32 ± 25.44	0.7	3590.13 ± 57.20	1.6
hydrochlorothiazide	25	25.88 ± 0.34	1.3	26.69 ± 1.29	4.8	25.45 ± 0.29	1.1	26.74 ± 3.13	11.7
	500	560.13 ± 0.70	0.1	517.28 ± 11.62	2.3	468.47 ± 1.66	0.4	531.65 ± 21.94	4.1
	5000	4719.25 ± 205.46	4.4	4739.30 ± 167.66	3.5	4700.72 ± 23.23	0.5	4745.30 ± 36.49	0.8
	5	5.46 ± 0.34	6.3	5.14 ± 0.06	1.2	4.87 ± 0.10	2.0	4.61 ± 0.09	2.0
nifedipine	100	100.30 ± 0.78	0.8	99.39 ± 1.34	1.4	97.56 ± 1.09	1.1	97.93 ± 1.18	1.2
	1000	873.99 ± 21.28	2.4	928.74 ± 16.08	1.7	927.25 ± 3.49	0.4	860.37 ± 22.96	2.7
	50	54.29 ± 0.94	1.7	49.32 ± 2.03	4.1	48.57 ± 1.40	2.9	52.12 ± 3.14	6.0
valsartan	1000	937.54 ± 19.41	2.1	901.96 ± 6.34	0.7	990.30 ± 7.76	0.8	1066.59 ± 68.31	6.4
	10000	9192.99 ± 167.34	1.8	9957.94 ± 963.19	9.7	8701.16 ± 60.77	0.7	8719.00 ± 100.69	1.2

**Table 6:** Pharmacokinetic parameters of the analytes in different groups (n = 6).

Compounds	C_max_ (ng/mL)	t_1/2_ (h)	AUC(0-tn) (h·ng/mL)	AUC(0-∞) (h·ng/mL)	T_max_ (h)	Ke (1/h)
metoprolol tartrate						
group A	1068.55 ± 330.39	0.84 ± 0.28	1621.21 ± 464.32	1628.06 ± 454.86	0.36 ± 0.15	0.90 ± 0.29
group B	2277.29 ± 240.30*∗∗*	1.06 ± 0.81	3787.97 ± 283.12*∗∗*	3829.12 ± 310.13*∗∗*	0.54 ± 0.25	0.88 ± 0.39
group C	3045.11 ± 424.22*∗∗*	2.16 ± 0.84*∗∗*	5975.49 ± 776.71*∗∗*	6020.93 ± 719.30*∗∗*	0.50 ± 0.00	0.42 ± 0.31*∗*
hydrochlorothiazide						
group A	1515.75 ± 235.11	3.26 ± 0.77	10091.55 ± 1285.15	10756.45 ± 1664.18	1.63 ± 1.24	0.23 ± 0.07
group B	2292.22 ± 209.37*∗∗*	2.68 ± 0.31	10687.69 ± 950.21	11020.65 ± 939.29	0.79 ± 0.19	0.26 ± 0.03
group C	1406.19 ± 248.74	2.52 ± 0.31	8479.60 ± 1243.01	8616.52 ± 1265.25*∗*	2.00 ± 0.00	0.28 ± 0.03
nifedipine						
group A	205.38 ± 73.57	4.45 ± 2.18	1914.09 ± 350.20	2090.41 ± 514.96	3.33 ± 1.03	0.20 ± 0.11
group B	554.60 ± 126.68*∗∗*	2.81 ± 0.85	4217.35 ± 1072.42*∗∗*	4289.90 ± 1100.45*∗∗*	3.33 ± 1.03	0.27 ± 0.08
group C	819.06 ± 146.92*∗∗*	3.12 ± 1.05	5706.47 ± 485.23*∗∗*	5810.56 ± 458.46*∗∗*	2.67 ± 1.51	0.25 ± 0.10
valsartan						
group A	3669.94 ± 720.62	2.97 ± 1.43	19563.78 ± 9850.62	21861.22 ± 12174.60	0.46 ± 0.29	0.27 ± 0.11
group B	3458.54 ± 324.28	2.47 ± 0.80	15146.64 ± 4814.24	17246.65 ± 6629.68	0.46 ± 0.10	0.31 ± 0.13
group C	4385.92 ± 1246.27	3.40 ± 0.88	20302.97 ± 6783.12	23030.31 ± 7186.54	0.24 ± 0.03	0.21 ± 0.04

## Data Availability

The data used to support the findings of this study are available from the corresponding author upon request.

## Supplementary Materials

Supplementary Materials The supplementary materials described the effect of Shuxuetong injection on the activity of CYP450 enzymes in rats. The results suggested that SXT had the potential to inhibit CYP3A4 and CYP2D6.
