# Supplementary material for: A High Throughput HPLC-MS/MS Method for Antihypertensive Drugs Determination in Plasma and Its Application on Pharmacokinetic Interaction Study with Shuxuetong Injection in Rats
Source: Biomed Res Int. 2019 Aug 6;2019:7537618. doi: 10.1155/2019/7537618 (PMC6699285; doi:10.1155/2019/7537618)
Supplement: Supplementary Materials — The supplementary materials described the effect of Shuxuetong injection on the activity of CYP450 enzymes in rats. The results suggested that SXT had the potential to inhibit CYP3A4 and CYP2D6. [file 7537618.f1.pdf]

## Optional Supplementary Materials

The purpose of this study was to evaluate the effect of SXT on the activity of CYP450 enzymes in rats. An LC-MS/MS method was established and validated for the simultaneous determination of two probe drugs including dapsone (CYP3A4) and dextromethorphan (CYP2D6) in rat plasma using irbesartan as internal standard (IS). The chromatographic separation was performed on a Waters XBridge$^{TM}$ C18 column (2.1 mm×100 mm, 3.5μm) using a gradient elution with the mobile phase consisting of acetonitrile and water (containing 0.1% formic acid) at a flow rate of 0.3 mL/min. The rats were randomly divided into three groups. A Group: the rats were given the solution of two probe drugs (0.5 mg/kg of dapsone, 1 mg/kg of dextromethorphan) through the tail vein. B Group: a single intravenous injection of SXT (0.625 mL/kg) and then dosing as the A group. C Group: injection of SXT (0.625 mL/kg) through the tail vein daily for 8 consecutive days and dosing as the A group on the eighth day. The pharmacokinetic results (Fig.R1, Table.R1) of the two probe drugs suggested that SXT had the potential to inhibit CYP3A4 and CYP2D6.

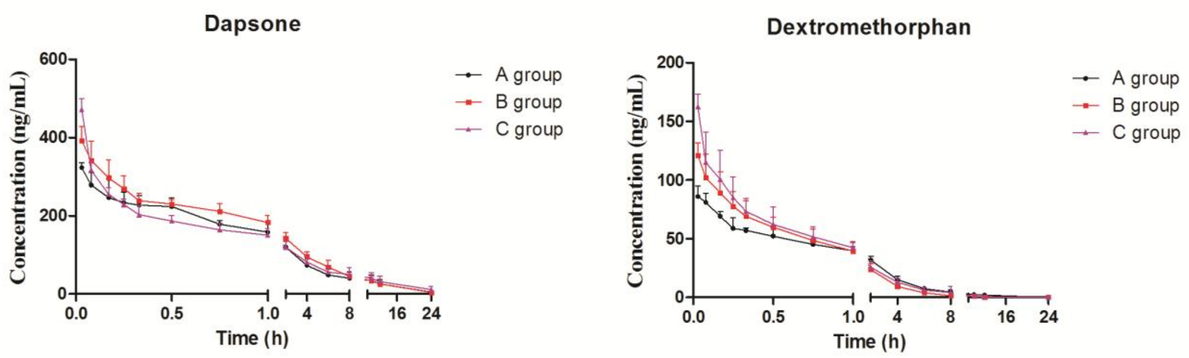


Fig.R1 Mean plasma concentration–time curves of probe drugs in different groups (n = 6)

Table.R1 Pharmacokinetic parameters of the probe drugs in different groups (n = 6).

| Probe drug and Group | $C_{max}$ (ng/mL) | $T_{1/2}$ (h) | $AUC_{(0-24)}$ (h ng/mL) | $AUC_{(0-\infty)}$ (h ng/mL) | $MRT_{(0-24)}$ (h) | $MRT_{(0-\infty)}$ (h) |
|---|---|---|---|---|---|---|
| dapsone | | | | | | |
| group A | 323.03 ± 12.51 | 1.84 ± 0.55 | 915.18 ± 48.52 | 951.00 ± 55.69 | 5.37 ± 0.58 | 6.29 ± 0.79 |
| group B | 392.42 ± 35.86** | 1.83 ± 0.56 | 1248.44 ± 197.98** | 1278.90 ± 209.60** | 5.08 ± 0.53 | 5.67 ± 0.72 |
| group C | 472.31 ± 26.90** | 0.76 ± 0.31** | 1221.02 ± 298.48** | 1367.43 ± 434.62** | 6.20 ± 1.04 | 8.89 ± 3.14 |
| dextromethorphan | | | | | | |
| group A | 86.15 ± 8.83 | 1.28 ± 0.27 | 98.29 ± 8.82 | 98.59 ± 8.75 | 3.15 ± 0.73 | 3.15 ± 0.73 |
| group B | 121.02 ± 10.60** | 0.62 ± 0.10** | 155.20 ± 34.23** | 156.39 ± 34.49** | 1.98 ± 0.40** | 2.23 ± 0.84* |
| group C | 162.61 ± 10.38** | 0.45 ± 0.09** | 187.17 ± 39.72** | 187.59 ± 40.26** | 2.43 ± 0.67 | 2.48 ± 0.78 |
